# Three-dimensional movement and distribution of *Tribolium castaneum* (Coleoptera: Tenebrionidae) and *Cryptolestes ferrugineus* (Coleoptera: Laemophloeidae) in stored wheat at different temperatures and at different times

**DOI:** 10.1093/ee/nvae130

**Published:** 2025-01-02

**Authors:** Harshini Boopathy, Vimala S K Bharathi, Digvir S Jayas, Fuji Jian

**Affiliations:** Department of Biosystems Engineering, University of Manitoba, Winnipeg, MB, Canada; Office of the President, University of Lethbridge, Lethbridge, AB, Canada; Department of Biosystems Engineering, University of Manitoba, Winnipeg, MB, Canada; Office of the President, University of Lethbridge, Lethbridge, AB, Canada; Department of Biosystems Engineering, University of Manitoba, Winnipeg, MB, Canada

**Keywords:** rusty grain beetle, red flour beetle, stored grain, 3-dimensional, distribution, temperature, movement period

## Abstract

Understanding the movement and distribution patterns of insects is crucial for developing effective stored grain management protocols. This research investigates 3-dimensional movement and distribution of *Tribolium castaneum* (Herbst) and *Cryptolestes ferrugineus* (Stephens) separately at different temperatures (5, 10, 20, and 30°C) and for different movement periods (1, 2, 3, and 24 h) in stored wheat with a uniform moisture content of 14.5% (wet basis). The experiments were conducted in a wooden container with internal dimensions of 0.7 × 0.7 × 0.7 m. The wheat was filled into 343 mesh cubes (referred to as cubes), each measuring 0.1 × 0.1 × 0.1 m, arranged in 7 layers after being loaded into the container. One hundred insects were introduced into the center cube (the center of the container) at the start of each experiment. After the desired movement period, the cubes were removed in less than 45 min from the wooden container. The wheat in each cube was wrapped in labeled plastic bags, sieved, and the insects were recovered and counted. Results indicated that both species exhibited movement speeds > 7.2 m/d in vertical and horizontal directions at higher temperatures (20 and 30°C). At lower temperatures (5 and 10°C), their vertical speed was higher than their horizontal speed. *Tribolium castaneum* ceased movement at 5°C, whereas ~13% of *C. ferrugineus* adults continued to move at this temperature. The drift effect and geotaxis influenced the movement and distribution of both species in the vertical direction, while their horizontal movement followed a diffusion pattern.

## Introduction

Canada stands as one of the world’s leading producers and exporters of wheat, with its vast agricultural landscape supporting a thriving grain industry (Statistics Canada 2023). The importance of grain storage systems in preserving the quality and safety of Canada’s wheat production cannot be understated. A zero-tolerance policy toward stored grain pests is maintained to recognize the importance of preventing infestations. Insect infestation presents a significant global challenge to grain storage facilities, endangering food security and economic stability. Grain storage environments provide a complex habitat for a wide range of insect species, offering shelter, food resources, and optimal conditions for reproduction. Insects such as weevils, beetles, and moths exhibit diverse movement behaviors that are influenced by factors like grain type, temperature, humidity, and structural features of storage facilities (Jian and Jayas 2012).

Among the damaging pests, *Cryptolestes ferrugineus* (Stephens) (Coleoptera: Laemophloeidae), commonly referred to as the rusty grain beetle and *Tribolium castaneum* (Herbst) (Coleoptera: Tenebrionidae), commonly known as the red flour beetle are recognized as the two major grain pests in Canada (Hulasare et al. 2003). These cosmopolitan beetles thrive in stored grain environments, causing extensive damage and contamination. The diet of *T. castaneum* primarily includes flour, broken grains, and other dried foods (Abdullahi et al. 2019). They are known to infest a wide range of commodities, such as spices, beans, and pasta. In contrast, the *C. ferrugineus* beetle primarily feeds on stored grains and other cereals (Hagstrum 2012). Adults of *T. castaneum* are 10 times larger than *C. ferrugineus* and are responsible for causing 15–20% more damage to grains (Jayas et al. 1995).

Insect movement and distribution pattern studies involve observing and analyzing how insects navigate, disperse, and establish populations within specific environments such as grain storage facilities. Different species exhibit varied behaviors and preferences for temperature, humidity, and grain type, which influence their movement patterns (Anukiruthika et al. 2021). By understanding these patterns, we can identify the key pathways and mechanisms through which pests infiltrate and spread within storage facilities. This knowledge is essential for developing effective pest management and control strategies to prevent infestations and preserve stored grains (Jian 2019).

Researchers have extensively studied *T. castaneum* and *C. ferrugineus* in controlled laboratory settings and field bins to understand their nature and behavior under various environmental conditions (Bharathi et al. 2023a, Chaubey 2023). A 1-dimensional (1-D) grain column was developed by Jian et al. (2004a) to study the movement of *C. ferrugineus* adults under both linear and dynamic temperature conditions and varying insect densities. The study examined insect behavior at different time intervals of 1, 3, 6, 12, 24, 72, and 144 h, with insect densities of 2, 12, 24, and 48 adults per kilogram of wheat (A/kg). In addition, this research was conducted across a wide range of temperatures, moisture gradients, and dockage percentages (Jian et al. 2003, 2005b). Because the initial study provided insights in only one direction, a 2-dimensional (2-D) grain chamber was developed to study horizontal movement in 2 directions (i.e., X and Y in the same plane) (Jian et al. 2007). This study revealed that *C. ferrugineus* adults follow a diffusion pattern at constant temperature and moisture levels within the 2-D grain chamber. Similarly, the horizontal and vertical movements of *T. castaneum* were also investigated within wheat and corn columns under constant and varying temperature gradients ranging from 20 to 30°C (Jian et al. 2005a). The first 3-dimensional (3-D) study was conducted by Surtees (1964), who used cube bags made of netting with openings to facilitate insect movement. The setup consisted of 64 cubes arranged in 4 layers. Insects were introduced at the top surface, and the cubes were removed at the end of the experiment. To address the limitations of this approach, such as the arrangement of bags for each experiment which led to a lack of consistency, and the restriction of movement examination to only the downward direction, Bharathi et al. (2021) modified the setup. Bharathi et al. (2022a) conducted a 24-h 3-D study on *C. ferrugineus* adults in wheat at constant temperatures (20 and 30°C) and moisture contents (12, 14, and 16% wet basis). In addition, another study by Bharathi et al. (2022b) examined the effects of various *C. ferrugineus* densities (0.35, 1.77, and 3.53 A/kg), movement periods (6, 24, and 72 h), and temperatures (20, 30, and 35°C). The results from these studies were consistent with previous 1-D and 2-D research, showing that the insects followed diffusion patterns and exhibited a drift effect in their distribution and movement.

Despite these studies, an extensive 3-D laboratory movement study of *T. castaneum* has not yet been reported. The temperature at which *C. ferrugineus* and *T. castaneum* adults cease to move within a 3-D setup remains unknown. This study extends the previous research by Bharathi et al. (2022a, 2022b) and aims to understand the movement and distribution of both *T. castaneum* and *C. ferrugineus* in a 3-D laboratory setup with 14.5% moisture of the wheat grain at different temperatures (5, 10, 20, and 30°C) and for different movement periods (1, 2, 3, and 24 h). Previous studies on *C. ferrugineus* insect movement did not include shorter movement periods, but such data are essential to develop a 3-dimensional (3-D) mathematical insect diffusion model combined with heat and mass transfer models, which typically requires time increments of 1–3 h.

## Materials and Methods

### Wheat

Around 2,000 kg of Canada Western Red Spring Wheat (Grade No.1, cv. AC Barrie, certified) was obtained from Richardson International Ltd., Winnipeg, Manitoba and utilized for this study. The wheat underwent cleaning procedures using a 3D shaker (Vibro-Energy Separator, SWECO, Florence, KY) to eliminate any dockage and it was stored in double-layered plastic bags. Subsequently, the wheat was stored at −15°C for a minimum of three weeks to ensure the absence of any insect contaminants (Fields and White 1997). The moisture content of the received wheat was determined by the oven drying method, where 10 g of wheat sample from each bag was dried at 130°C for 19 h (ASABE 2022). The moisture content of the samples was 11 ± 0.5% (wet basis). The wheat was conditioned to the desired moisture content of 14.5% (wet basis) by adding the desired amount of distilled water. The mixture was then rotated in the mixer for ~35–40 min. The conditioned wheat was again stored in double-layered plastic bags at 10°C for 2–4 wk for moisture equilibration. At least 4 d before the experiment, the wheat bags were transferred to an environmental chamber (CMP 4030, Controlled Environments Limited, Winnipeg, MB), maintaining the experimental temperature. The moisture content of conditioned wheat was verified by the oven drying method. This procedure was repeated for each experiment.

### Experimental Setup

**Fig. 1.**
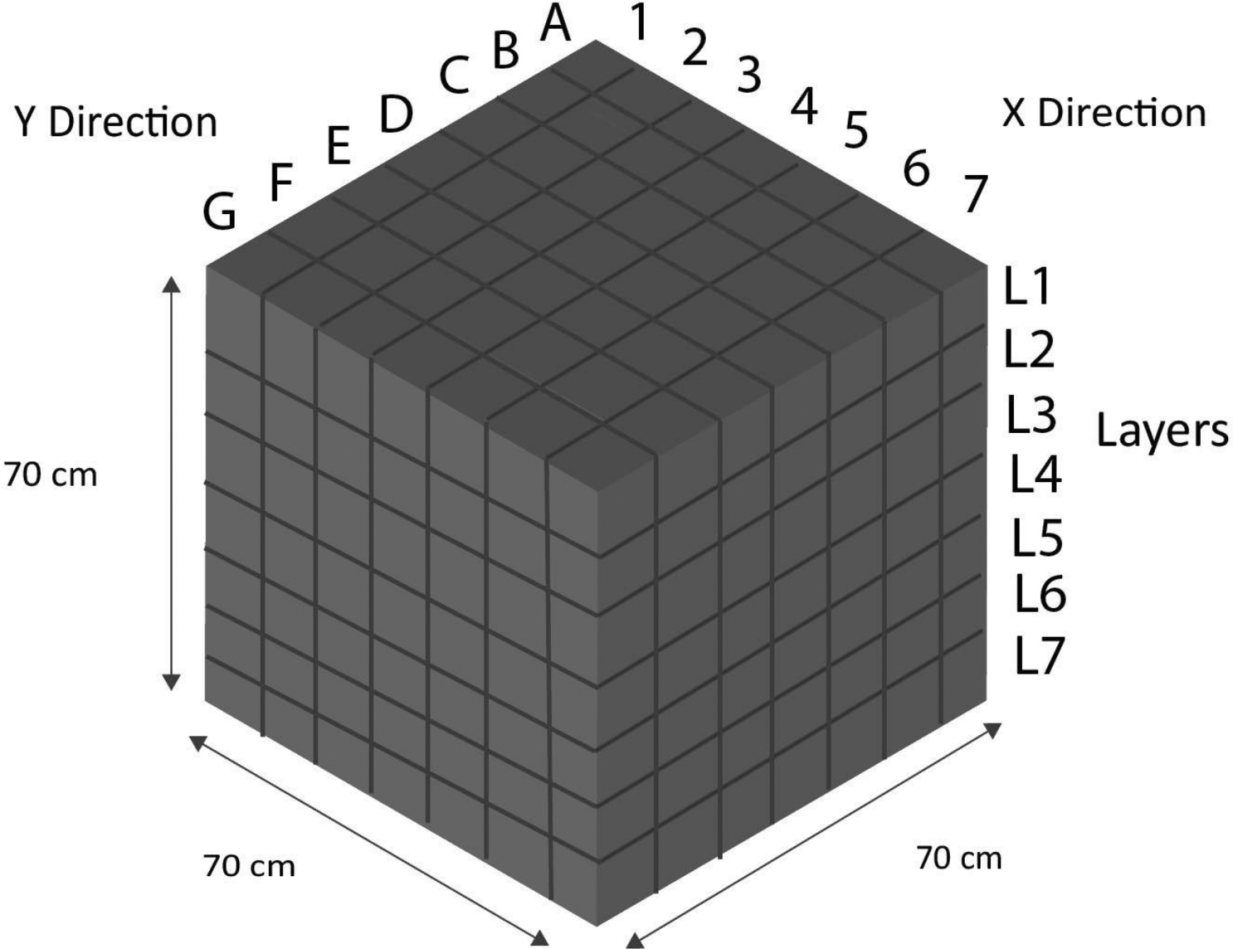
Internal dimensions of the wooden container and numeration of each small mesh cube. Insects were introduced in the L4-D-4 cube located at the center.

The experimental setup was the same as that utilized by Bharathi et al. (2022b) with a modification to the fabrication of mesh cube frames. A total of 343 mesh cubes, each measuring 0.1 × 0.1 × 0.1 m were arranged within a wooden container with internal dimensions of 0.7 × 0.7 × 0.7 m (Fig. 1). The mesh cubes were constructed using 1.6 mm diameter metal rods which were framed to a single cube. These mesh cubes were then enclosed by cloth screens with openings of 1.4 × 1.4 mm, which enabled insect movement between cubes while preventing wheat from escaping the cubes (Bharathi et al. 2021). One day before the experiment, all the cubes were filled with conditioned wheat, a process that took ~2–3 h. The mesh cubes were then loaded into the wooden container layer by layer to form aligned rows and columns (Fig. 1). The wooden container was then sealed with a lid and a double-sided tape was applied along the top edges.

### Insects

Both *C. ferrugineus* and *T. castaneum* adults were cultured in a laboratory environment maintained at 30 ± 1°C and 75 ± 5% relative humidity. For *C. ferrugineus*, a medium consisting of a mixture of whole wheat kernels, cracked wheat, and wheat germ in a mass ratio of 90:5:5 was utilized. Meanwhile, *T. castaneum* was reared on a mixture of wheat flour and brewer’s yeast in a mass ratio of 95:5. For all the experiments, adults of insects aged between 1 d and 2 mo were utilized. One day before each experiment, 100 adults were sieved from the laboratory culture and placed inside a mesh cube for acclimation. This cube contained the same wheat as used for the experiments. Subsequently, the cube was positioned inside a plastic container (33.1 × 23.5 × 17.3 cm) for 24-h acclimation within the environmental chamber (Conviron CMP1010, Controlled Environments Ltd., Winnipeg, MB, Canada) set at the experimental temperature (5, 10, 20, or 30°C). For the 5°C experiment with *C. ferrugineus*, a 120-h acclimation period was implemented during which the temperature was gradually reduced at a rate of 5°C/d, while a 24-h acclimation period was used for all other temperatures.

### Experimental Procedure

A day before the insect movement test, the prepared wheat was filled into the mesh cubes and placed inside the wooden container, except for the cube containing adults, which was placed inside a plastic container. Each mesh cube was labeled for convenience, with the × direction numbered from 1 to 7, the y direction from A to G, and the layers from L1 to L7 (Fig. 1). The first cube at the top was labeled as L1-A-1, while the center cube containing the adults was labeled as L4-D-4. After 24-h acclimation period, on the day of the insect movement test, the cube containing the adults was placed at the L4-D-4 location by removing only the center cubes from the top three layers. Cubes were packed and there were no gaps among cubes. The wooden container was then covered with a plywood lid and sealed with a double-sided tape. After the desired movement period, the lid was opened, and the cubes were removed. Each cube was placed into a pre-labeled plastic bag according to its position and each bag was tied, to prevent any escaping insects. This process took ~30–45 min in total and was conducted inside the environmental chamber.

Adults in each labeled cube were separated using a shaker with a No. 14 sieve (1.4 mm mesh openings). Not all introduced adults were successfully recovered, as some adults tended to hide among the wheat kernels. Some adults were stuck on the tapes which were used to seal the lid of the wooden container. Out of 100 introduced *T. castaneum* adults, 1 to 6 individuals were recovered from the tape for all the tests conducted at 20 and 30°C while no adults were recovered from the tape at 10 and 5°C. In addition, no adults of *C. ferrugineus* were recovered from the tape under any of the tested conditions.

The mean recovery rate was 93.2% (*n* = 19) for *T. castaneum* and 97.4% (*n* = 18) for *C. ferrugineus*. To account for the missed adults, each replicate’s total number of recovered insects was normalized using Eq. 1 (Jian et al. 2007).


$$\begin{array}{l} \begin{array}{l} Number\;of\;adults\;in\;the\;cube\;=\;\frac{Number\;of\;adults\;recovered\;from\;the\;cube\;\times \;100}{Total\;number\;of\;adults\;recovered\;from\;all\;cubes}\;\;\;\; \end{array} \end{array}$$ (1)

where 100 represents the number of initially introduced adults.

### Statistical Analysis

A total of 14 experiments, consisting of 7 experiments for *T. castaneum* and 7 experiments for *C. ferrugineus* were conducted. The tests were conducted with 14.5% moisture content wheat at 5, 10, 20, and 30°C for movement periods of 1, 2, 3, and 24 h. Each of the 7 experiments was replicated three times, except the experiment for *T. castaneum* at 5°C, which was conducted only once due to the absence of insect movement. The data used for analyzing and comparing the 24-h movement of *C. ferrugineus* at 30°C was sourced from Bharathi et al. (2022a).

An ANOVA followed by a Tukey’s test was conducted to assess whether the adults exhibit any preference for specific layers within the setup and to determine if this preference changes with variations in environmental conditions. Bartlett’s test was performed prior to ANOVA to evaluate the assumptions of homoscedasticity. The test was carried out within different layers for each experimental condition and within each layer across various experiments, all at a 0.05 confidence level. To address the risk of Type I errors associated with multiple comparisons, the Tukey Honestly Significant Difference (HSD) test was employed, ensuring that the confidence level remains at 0.05 during all the comparisons (Greenwood and Banner 2015, Navarro 2019).

**Fig. 2.**
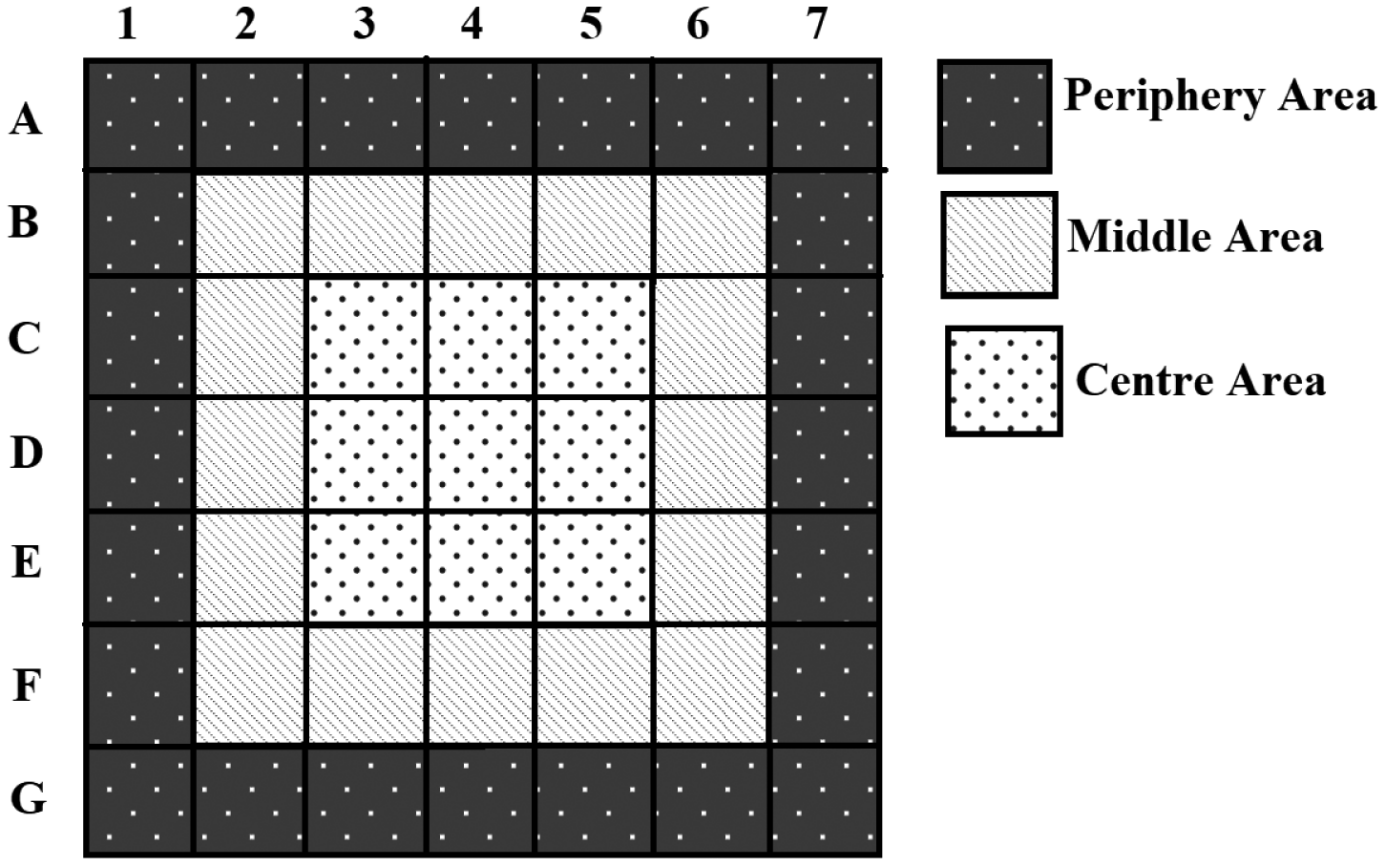
Categorization and numeration of mesh cubes inside the wooden container


To examine whether adults exhibit a preference for the boundary or the center under each experimental condition, each layer was subdivided into three categories: center, middle, and periphery (Fig. 2). The adult density in each area was computed (Eq. 2), and Tukey test was performed.

$$\begin{array}{l} \begin{array}{l} Adult\;density\;=\;\;\frac{Number\;of\;adults\;in\;the\;area}{Total\;wheat\;mass\;in\;the\;area}\; \end{array} \end{array}$$ (2)

A 2-sample location test and Empirical Distribution Function (EDF) statistics were performed using R Studio software (version 4.3.1) to analyze whether adults exhibit similar movement patterns in both horizontal and vertical directions under different temperatures and movement periods (Jian et al. 2002). This analysis involved comparing two temperatures in the same movement period (1 h) and two movement periods at the same temperature (30°C). The variations in each layer were assessed by the Wilcoxon test, while the cumulative distribution along the vertical direction was analyzed using Kolmogorov–Smirnov test and a Mood’s median test was conducted to analyze the general movement of the introduced adults. To control the table-wise Type I error rate during multiple comparisons, a sequential Holm–Bonferroni test was applied to each group. All the analyses were performed separately for both *T. castaneum* and *C. ferrugineus* adults.

To calculate the maximum vertical movement speed of the adults at different temperatures, the distance traveled by the fast-moving adult in both the upward and downward directions from the point of introduction within 1 h at ≥ 10°C and 24 h at 5°C was calculated. Since the point of the introduction is in the middle of the wooden container, the distances traveled to the upward layers 1, 2, and 3 (Fig. 1) were 30, 20, and 10 cm, respectively. In the downward direction, the distances traveled to layers 5, 6, and 7 (Fig. 1) were 10, 20, and 30 cm, respectively. The maximum movement speed in the horizontal direction was the distance from the point of introduction to the location where the furthest-moving adults were recovered, divided by the movement time. The distance from the center, middle, and periphery area were 10, 20, and 30 cm, respectively (Fig. 2).

## Results

### Movement and Distribution of *Tribolium castaneum* Adults

#### Movement in the Vertical Direction

**Fig. 3.**
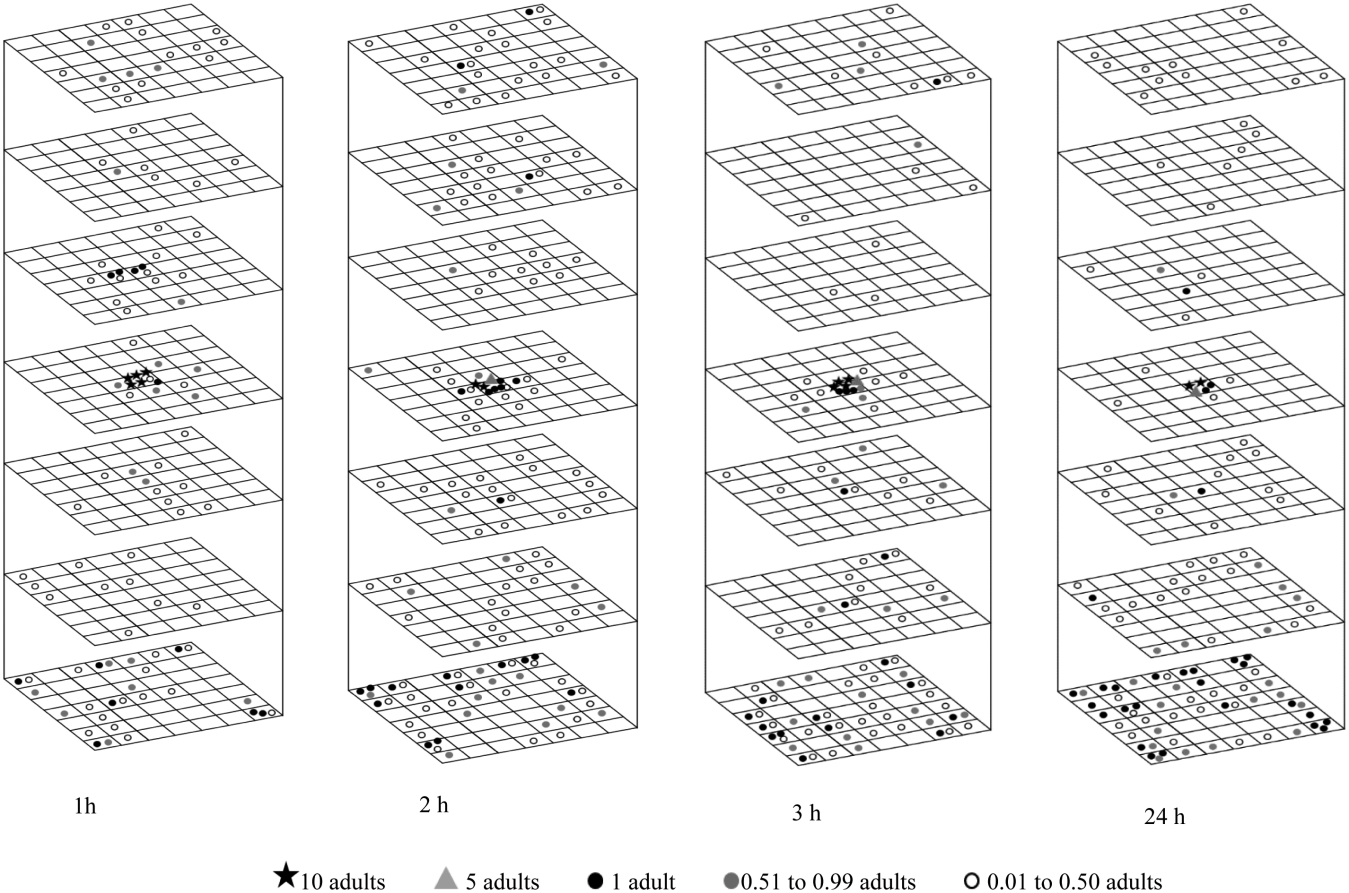
Redistribution of 100 *Tribolium castaneum* adults at 30°C in wheat with 14.5% moisture content in different movement periods (following a 24-h acclimation period). The symbols represent the mean insect number for three replications.

**Fig. 4.**
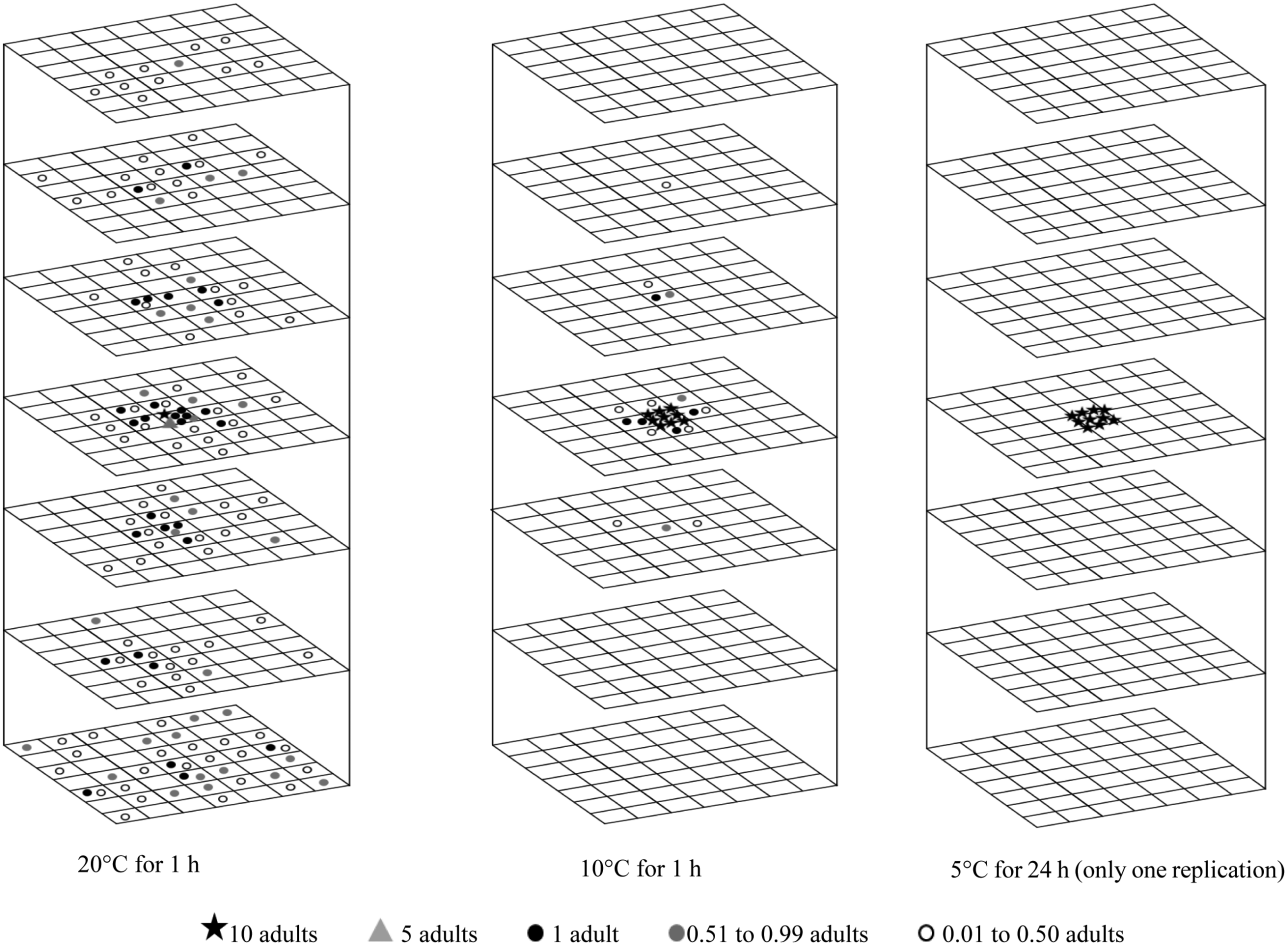
Redistribution of 100 *Tribolium castaneum* adults in wheat with 14.5% moisture content at 20, 10, and 5°C (following a 24-h acclimation period). The symbols represent the mean insect number for three replications, except at 5°C for 24 h.

At 20 and 30°C, the adults of *T. castaneum* displayed active movement in both upward and downward directions, and more adults moved down (Figs. 3–4). When the temperature was reduced to 10°C, these adults restricted their movement, and more adults moved up (Fig. 4). As the temperature was further decreased to 5°C, the adults ceased movement (Fig. 4). The adults moved at a speed of more than 7.2 m/d at 30 and 20°C in both upward and downward vertical directions. This speed decreased to 4.8 m/d in the upward direction and 2.4 m/d in the downward direction at 10°C (Table 1).

**Table 1.** Maximum movement speed of *Tribolium castaneum* and *Cryptolestes ferrugineus* in vertical and horizontal directions

Insect	Grain temperature (°C)	Movement speed (m/d)		
		Vertical direction		Horizontal direction
		Upward direction	Downward direction	
*Tribolium castaneum*	**30**	>7.2	>7.2	>7.2
	**20**	>7.2	>7.2	>7.2
	**10**	4.8	2.4	2.4
*Cryptolestes ferrugineus*	**30**	>7.2	>7.2	>7.2
	**20**	>7.2	>7.2	>7.2
	**10**	2.4	>7.2	4.8
	**5**	0.2	0.2	0.1

As the movement duration increased from 1 to 2 h at 30°C, the upward movement increased from 19 to 22%. However, when further increasing time to 3 and 24 h, more adults moved down. For example, only 8% and 9% of adults moved upward in 3 and 24 h, respectively. At 20 and 30°C, the mean fraction of recovered adults in the bottom layer was notably higher compared to the other layers. At 20 and 30°C in 1 h, 21.1 and 16.9% of adults were recovered in the bottom layer, respectively. At 30°C and with the time increased from 1 to 24 h, the recovered adults in the bottom layer were increased from 16.9 to 42.3% (Table 2). The Tukey test also showed the numbers in the bottom layer and the insect introduction layer were significantly higher than other layers at both 30 and 20°C (Table 2). The 2-sample location test and EDF statistics revealed that the adults at 20 and 30°C had significantly different distribution patterns (Table 3). When the temperature was at 30°C, the adults gradually shifted to a downward movement after an initial upward movement.

**Table 2.** Recovered adults of *Tribolium castaneum* in different layers at each tested condition

Layer		Tested condition^γ^					
		30°C ^α^; 1 h ^λ^	30°C ^α^; 2 h ^λ^	30°C ^α^; 3 h ^λ^	30°C ^α^; 24 h ^λ^	20°C ^α^; 1 h ^λ^	10°C ^α^; 1 h ^λ^
1		7.9 ± 2.3 ^b δ^	9.6 ± 6.6 ^bc A^	5.1 ± 3.7 ^b A^	4.1 ± 1.3 ^b A^	4.4 ± 1.0 ^b δ^	0.0 ± 0.0 ^c ψ^
2		2.9 ± 3.3 ^b δ^	8.0 ± 4.3 ^c A^	1.8 ± 1.7 ^b A^	2.2 ± 3.0 ^b A^	8.1 ± 6.6 ^b δ^	0.3 ± 0.6 ^bc δ^
3		8.3 ± 4.2 ^b δ ψ^	4.4 ± 3.2 ^c A^	1.1 ± 2.0 ^b A^	3.0 ± 0.7 ^b A^	12.1 ± 5.2 ^b δ^	2.0 ± 1.0 ^b ψ^
4		55.8 ± 15.1 ^a ψ^	36.1 ± 4.2 ^a A^	53.0 ± 23.5 ^a A^	31.6 ± 9.8 ^a A^	32.6 ± 11.9 ^a ψ^	96.3 ± 0.6 ^a δ^
5		4.7 ± 1.6 ^b δ ψ^	7.7 ± 1.3 ^c A^	5.6 ± 4.8 ^b A^	5.6 ± 2.2 ^b A^	13.4 ± 7.2 ^b δ^	1.3 ± 1.1 ^bc ψ^
6		3.6 ± 0.6 ^b δ ψ^	8.9 ± 4.2 ^c A^	6.8 ± 3.5 ^b A^	11.2 ± 5.1 ^b A^	8.4 ± 3.5 ^b δ^	0.0 ± 0.0 ^c ψ^
7		16.9 ± 11.8 ^b δ ψ^	25.3 ± 10.8 ^ab A^	26.6 ± 18.7 ^ab A^	42.3 ± 11.3 ^a A^	21.2 ± 6.9 ^ab δ^	0.0 ± 0.0 ^c ψ^
ANOVA test values	***F***	18.590	12.920	8.035	20.570	6.047	9080
	***P***	<0.001	<0.001	0.001	<0.001	0.003	<0.001

^γ^The lowercase letters a, b, and c in a column indicate significantly different mean values among different layers under the same tested condition. Letters A, B, and C in a row indicate significantly different mean values among the same layers at 30°C in 2, 3, and 24 h movement periods. Symbols δ, ψ, and ϕ in a row, indicate significantly different mean values among the same layers in 1 h at 10, 20, and 30°C. Tukey’s test at a 0.05 confidence level was used to conduct these comparisons. Degrees of freedom (df) = 6 for each column comparisons, and df = 2 for each comparison in a row.

^α^Grain temperature;

^λ^movement period.

**Table 3.** Two-sample location test and Empirical Distribution Function (EDF) statistics result for *Tribolium castaneum* movement and distribution in the vertical direction

Experimental condition^γ^		Wilcoxon^λ^		Kolmogorov–Smirnov^λ^		Mood median^λ^	
Temperature (°C)	Time (h)	*Z*	*P*	*D*	*P*	*Z*	*P*
30	1 vs 2	4.420	<0.001***	−0.548	<0.001***	1.741	0.082
30	2 vs 3	4.145	<0.001***	−0.571	<0.001***	3.539	<0.001***
30	3 vs 1	3.207	0.001**	−0.405	<0.001***	2.915	0.003**
30	24 vs 1	3.920	<0.001***	−0.405	<0.001***	1.575	0.115
30	24 vs 2	4.858	<0.001***	−0.571	<0.001***	2.201	0.028*
30	24 vs 3	3.570	<0.001***	−0.429	<0.001***	3.210	0.001**
30 vs 20	1	4.870	<0.001***	−0.524	<0.001***	1.139	0.255
30 vs 10	1	0.663	0.507	−0.262	0.041*	4.796	<0.001***
20 vs 10	1	2.251	0.024*	−0.405	<0.001***	5.723	<0.001***

^γ^Comparison between two experiments.

^λ^Values from the Wilcoxon, Kolmogorov–Smirnov, and median tests. * Significant was at the table-wise level adjusted using the Holm–Bonferroni method. During the Holm–Bonferroni test, the first six *P* values, and the last three *P* values were categorized into two groups, and comparisons were conducted separately.

#### Movement in the Horizontal Direction

The adult movement in the horizontal direction increased with the increase of both temperature and movement period, and a diffusion pattern in the horizontal direction was observed across nearly all layers (Figs. 3–4). At temperatures of 30 and 20°C, the adults moved with a movement speed of more than 7.2 m/d in the horizontal direction, which was reduced to 2.4 m/d at 10°C (Table 1) At the temperature of 30°C and with extended movement periods, the adults in the bottom layer (layer 7) tended to accumulate at the periphery of the container (Fig. 3). Although the adults in the introduced layer (layer 4) moved upward and downward over time, a high density of adults remained at the center location, indicating that many adults did not move and stayed within their introduced location under all tested conditions (Figs. 3 and 4, Table 4). The statistical analysis confirmed these observations (Table 4). For instance, at 30°C, 4.1–7.2% of adults consistently remained in the center location of the introduced layer (layer 4) rather than the middle and periphery locations during various movement periods (Table 4). Therefore, adults did not move or moved both upward and downward from their introduced location, exhibiting a diffusion pattern in the horizontal direction. Their movement was constrained by the boundary of the wooden container, leading them to remain near the periphery in the bottom layer (layer 7).

**Table 4.** Recovered densities of *Tribolium castaneum* adults in different areas in the same layer at the same tested conditions

Layer	Time (h)^λ^	Center	Middle	Periphery	Tukey test values	
					*F*	*P*
30°C^µ^						
1^γ^	1	0.2 ± 0.2 ^a^	0.2 ± 0.1 ^a^	0.1 ± 0.1 ^a^	0.424	0.673
	2	0.3 ± 0.3 ^a^	0.2 ± 0.2 ^a^	0.2 ± 0.1 ^a^	0.377	0.701
	3	0.3 ± 0.2 ^a^	0.0 ± 0.1 ^a^	0.2 ± 0.1 ^a^	1.717	0.257
	24	0.1 ± 0.1 ^a^	0.1 ± 0.1 ^a^	0.1 ± 0.1 ^a^	0.726	0.522
2^γ^	1	0.2 ± 0.3 ^a^	0.0 ± 0.1 ^a^	0.1 ± 0.1 ^a^	1.361	0.326
	2	0.4 ± 0.2 ^a^	0.2 ± 0.2 ^a^	0.1 ± 0.1 ^a^	2.299	0.182
	3	0 ^a^	0.0 ± 0.4 ^a^	0.1 ± 0.1 ^a^	1.317	0.336
	24	0.1 ± 0.2 ^a^	0.3 ± 0.1 ^a^	0.1 ± 0.1 ^a^	0.274	0.769
3^γ^	1	0.8 ± 0.5 ^a^	0.1 ± 0.1 ^b^	0.1 ± 0.0 ^ab^	6.363	0.033
	2	0.3 ± 0.2 ^a^	0.1 ± 0.1 ^a^	0.3 ± 0.1 ^a^	3.792	0.086
	3	0.1 ± 0.1 ^a^	0.1 ± 0.1 ^a^	0 ^a^	0.503	0.628
	24	0.3 ± 0.0 ^a^	0 ^b^	0.0 ± 0.1 ^b^	213.500	<0.001
4^γ^	1	7.2 ± 2.2 ^a^	0.1 ± 0.1 ^b^	0.0 ± 0.1 ^b^	32.870	<0.001
	2	4.5 ± 0.4 ^a^	0.1 ± 0.1 ^b^	0.1 ± 0.1 ^b^	429.500	<0.001
	3	7.0 ± 3.4 ^a^	0.1 ± 0.2 ^b^	0 ^b^	11.720	0.008
	24	4.1 ± 1.5 ^a^	0.0 ± 0.1 ^b^	0.0 ± 0.1 ^b^	23.010	0.002
5^γ^	1	0.4 ± 0.3 ^a^	0.0 ± 0.1 ^a^	0.1 ± 0.1 ^a^	3.567	0.095
	2	0.4 ± 0.2 ^a^	0.1 ± 0.1 ^a^	0.2 ± 0.0 ^a^	4.192	0.073
	3	0.3 ± 0.5 ^a^	0.1 ± 0.1 ^a^	0.1 ± 0.2 ^a^	0.694	0.536
	24	0.3 ± 0.2 ^a^	0.1 ± 0.1 ^a^	0.1 ± 0.0 ^a^	2.972	0.127
6^γ^	1	0.1 ± 0.2 ^a^	0.0 ± 0.1 ^a^	0.1 ± 0.1 ^a^	1.250	0.352
	2	0.2 ± 0.3 ^a^	0.2 ± 0.2 ^a^	0.3 ± 0.1 ^a^	0.285	0.762
	3	0.4 ± 0.4 ^a^	0.1 ± 0.0 ^a^	0.1 ± 0.1 ^a^	1.247	0.353
	24	0.1 ± 0.1 ^a^	0.1 ± 0.1 ^a^	0.5 ± 0.3 ^a^	5.128	0.051
7^γ^	1	0.3 ± 0.2 ^a^	0.1 ± 01 ^a^	0.6 ± 0.6 ^a^	1.455	0.305
	2	0.3 ± 0.4 ^a^	0.4 ± 0.2 ^a^	0.9 ± 0.5 ^a^	2.441	0.168
	3	0.5 ± 0.4 ^a^	0.4 ± 0.3 ^a^	0.9 ± 0.6 ^a^	0.801	0.492
	24	0.5 ± 0.5 ^b^	0.6 ± 0.1 ^ab^	1.6 ± 0.3 ^a^	7.983	0.020
20°C^µ^						
^1γ^	1	0.3 ± 0.1 ^a^	0.1 ± 0.1 ^ab^	0.0 ± 0.1 ^b^	5.578	0.043
2^γ^	1	0.7 ± 0.7 ^a^	0.1 ± 0.1 ^a^	0.1 ± 0.1 ^a^	2.198	0.192
3^γ^	1	1.1 ± 0.5 ^a^	0.2 ± 0.1 ^b^	0.1 ± 0.0 ^a^	12.170	0.008
4^γ^	1	3.4 ± 1.3 ^a^	0.2 ± 0.1 ^b^	0.1 ± 0.1 ^b^	22.950	0.002
5^γ^	1	1.2 ± 0.8 ^a^	0.3 ± 0.1 ^a^	0.1 ± 0.1 ^a^	4.796	0.057
6^γ^	1	0.5 ± 0.3 ^a^	0.2 ± 0.0 ^a^	0.1 ± 0.0 ^a^	4.377	0.067
7^γ^	1	0.7 ± 0.1 ^a^	0.5 ± 0.2 ^a^	0.5 ± 0.3 ^a^	0.832	0.480

^**µ**^ Grain temperature.

^**λ**^ Movement period.

^**γ**^ The alphabets a and b in the same row indicate significantly different mean values in a layer under the same tested condition.

Tukey’s test at a 0.05 confidence level was used to conduct these comparisons with degrees of freedom (df) = 2 for each row comparison.

### Movement and Distribution of *Cryptolestes ferrugineus*

#### Movement in the Vertical Direction

**Fig. 5.**
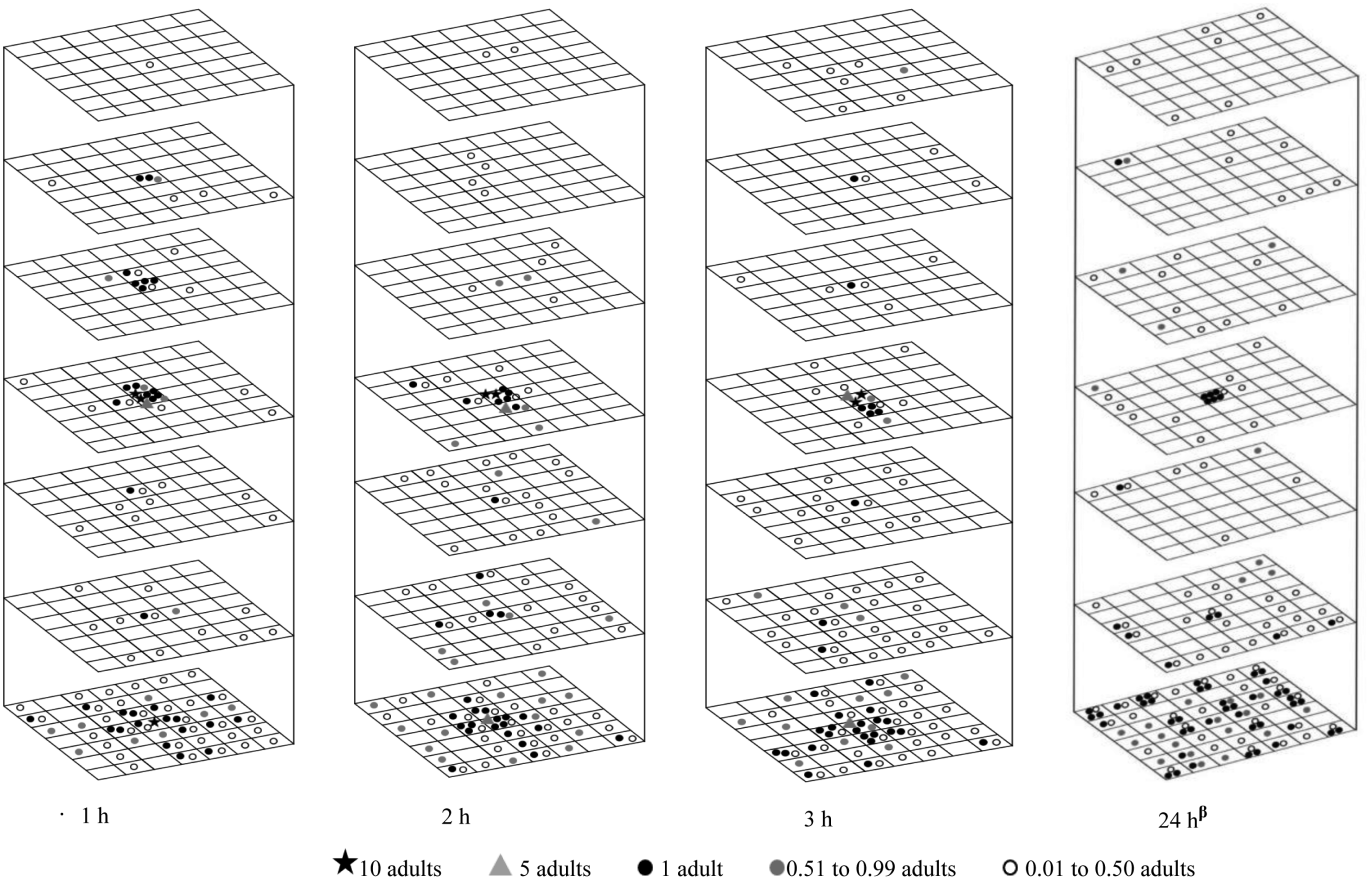
Redistribution of 100 *Cryptolestes ferrugineus* adults at 30°C in wheat with 14.5% moisture content for a movement period of 1 h to 24 h (following a 24 h acclimation period). Note: β Image used from Bharathi et al. (2022a). The symbols represent the mean insect number for three replications.

**Fig. 6.**
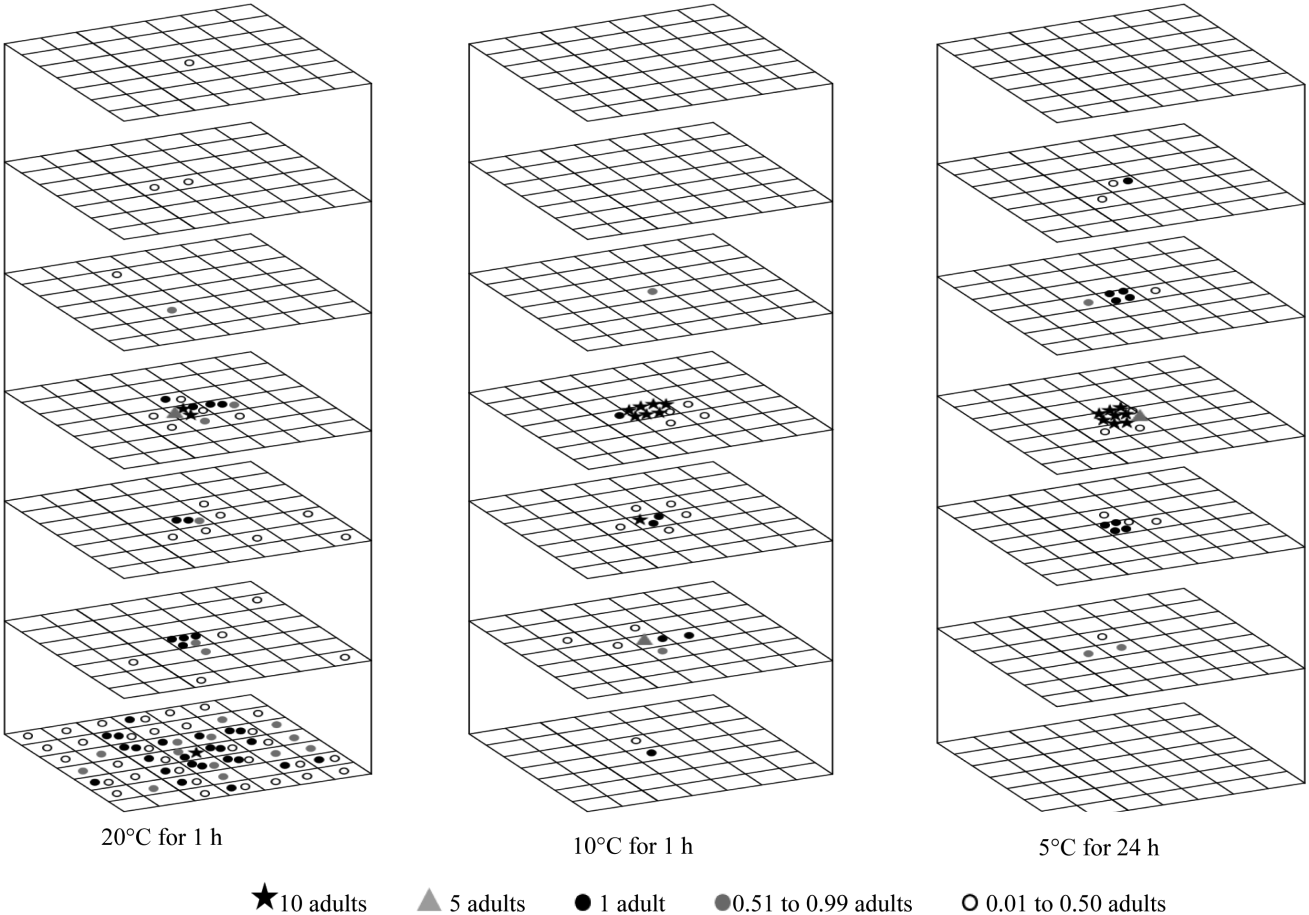
Redistribution of 100 *Cryptolestes ferrugineus* adults after1 h movement period in wheat with 14.5% moisture content at 20 and 10°C (following a 24-h acclimation period), and after 24 h at 5°C (following a 5 d acclimation period with gradual temperature reduction). The symbols represent the mean insect number over three replications.

*Cryptolestes ferrugineus* adults exhibited a clear preference for downward movement over upward movement in the vertical direction. Their movement increased with rising temperatures. At 30°C, 79% moved downward, and at 5°C, only 6% of adults moved downwards (Figs. 5-6). As the duration increased from 1 to 24 h at 30°C, the adult’s downward movement increased from 54% to 79% (Fig. 5). At 20 and 30°C, their movement speed exceeded 7.2 m/d in both directions. However, as the temperature decreased to 10°C, the upward movement speed reduced to 2.4 m/d while the downward movement speed remained above 7.2 m/d. At 5°C, the movement speed in both directions was reduced to 0.2 m/d (Table 1).

The bottom layer consistently showed the highest mean fraction of recovered insects compared to the other layers under all tested conditions except at 10 and 5°C (Fig. 6. Table 5). At 5°C, the mean fraction of adults recovered in the introduced layer was high (87%), but this recovery gradually decreased to 10% as the temperature increased to 30°C. At 20 and 30°C, the 2-sample location test and EDF statistics revealed significant variations, indicating that adults exhibited different movement patterns over time. This demonstrates that adults did not display consistent movement patterns at 20 and 30°C (Table 6). Furthermore, a noticeable shift in the adult distribution was observed at 10°C (Table 6). This indicates that higher temperatures (20 and 30°C) facilitate greater movement and dispersal in the vertical direction, while lower temperatures restrict movement.

**Table 5.** Recovered adults of *Cryptolestes ferrugineus* in different layers for each tested condition

Layer		Tested condition^γ^						
		30°C ^α^; 1 h^λ^	30°C ^α^; 2 h^λ^	30°C ^α^; 3 h^λ^	30°C ^α^; 24 h^λ^^β^	20°C ^α^; 1 h^λ^	10°C ^α^; 1 h^λ^	5°C ^α^; 24 h^λ^
1		0.4 ± 0.6 ^b δ^	0.7 ± 1.3 ^b A^	2.7 ± 0.6 ^b A^	2.4 ± 1.6 ^b A^	0.3 ± 0.6 ^b δ^	0 ^b δ^	0 ^b^
2		3.8 ± 2.2 ^b δ^	1.4 ± 1.7 ^b A^	2.0 ± 1.8 ^b A^	3.8 ± 4.9 ^b A^	0.7 ± 1.2 ^b δ ψ^	0 ^b ψ^	1.7 ± 2.0 ^b^
3		6.9 ± 3.0 ^b δ^	2.9 ± 3.4 ^b A^	3.1 ± 1.0 ^b A^	4.8 ± 3.2 ^b A^	1.0 ± 1.0 ^b ψ^	0.7 ± 10.2 ^b ψ^	5.0 ± 4.0 ^b^
4		35.2 ± 15.4^a δ^	36.3 ± 12.8^a A^	32.7 ± 14.2^a A^	10.0 ± 4.2^b A^	41.8 ± 17.3^a δ^	73.7 ± 17.9^a δ^	86.9 ± 7.5 ^a^
5		4.2 ± 2.6 ^b δ^	6.9 ± 3.2 ^b A^	5.5 ± 4.3 ^b A^	3.5 ± 2.2 ^b A^	5.5 ± 3.2 ^b δ^	14.6 ± 8.2 ^b δ^	5.1 ± 3.5 ^b^
6		5.9 ± 3.2 ^b δ^	11.8 ± 9.6 ^b A^	11.6 ± 8.8 ^b A^	15.1 ± 9.9^b A^	6.5 ± 3.6 ^b δ^	9.1 ± 10.6 ^b δ^	1.7 ± 0.6 ^b^
7		43.7 ± 19.5^a δ^	39.9 ± 4.0 ^a B^	42.3 ± 5.8 ^a B^	60.4 ± 7.9^a A^	44.3 ± 17.4^a δ^	2.0 ± 1.0 ^b ψ^	0 ^b^
ANOVA test values	***F***	9.874	19.640	16.900	41.700	13.1700	29.950	239.400
	***P***	<0.001	<0.001	<0.001	<0.001	<0.001	<0.001	<0.001

^β^Data used from Bharathi et al. (2022a).

^γ^The lowercase letters a, b, and c in a column indicate significantly different mean values among different layers under the same tested condition. Letters A, B, and C in a row indicate significantly different mean values among the same layers at 30°C in 2, 3, and 24 h movement periods. Symbols δ, ψ, and ϕ in a row, indicate significantly different mean values among the same layers in 1 h at 10, 20, and 30°C. Tukey’s test at a 0.05 confidence level was used to conduct these comparisons. Degrees of freedom (df) = 6 for each column comparison, and df = 2 for each comparison in a row.

^α^Grain temperature;

^λ^Movement period.

**Table 6.** Two-sample location test and Empirical Distribution Function (EDF) statistics results for *Cryptolestes ferrugineus* movement and distribution in the vertical direction

Experimental condition^γ^		Wilcoxon^λ^		Kolmogorov–Smirnov^λ^		Mood median^λ^	
Temperature (°C)	Time (h)	Z	*P*	*D*	*P*	*Z*	*P*
30	1 vs 2	3.520	<0.001***	−0.452	<0.001***	4.022	<0.001***
30	2 vs 3	3.732	<0.001***	−0.452	<0.001***	3.415	<0.001***
30	3 vs 1	3.769	<0.001***	−0.429	<0.001***	2.441	0.0145*
30	24 vs 1	3.769	<0.001***	−0.429	<0.001***	2.894	0.004**
30	24 vs 2	3.657	<0.001***	−0.452	<0.001***	3.856	<0.001***
30	24 vs 3	3.957	<0.001***	−0.429	<0.001***	2.273	0.023*
30 vs 20	1	2.782	0.005**	−0.405	<0.001***	4.359	<0.001***
30 vs 10	1	1.719	0.086	−0.357	0.003**	4.749	<0.001***
20 vs 10	1	0.769	0.442	−0.333	0.006**	5.505	<0.001***
5 vs 30	24	1.857	0.063	−0.333	0.005**	4.725	<0.001***

^γ^Comparison between two experiments, with the first column representing the constant experimental condition and the second column representing the variables being compared.

^λ^Values from the Wilcoxon, Kolmogorov–Smirnov, and median tests. * Significant was at the table-wise level adjusted using the Holm–Bonferroni method. During the Holm–Bonferroni test, the first 6 *P* values and the next 3 *P* values were categorized into two groups, and comparisons were conducted separately.

#### Movement in the Horizontal Direction

An increase in temperature and movement period resulted in greater diffusion of *C. ferrugineus* adults (Figs. 5 and 6). The adults moved horizontally at speeds more than 7.2 m/d at 30°C and 20°C, but this speed decreased to 4.8 m/d at 10°C and further reduced to 0.1 m/d at 5°C (Table 1). At 30°C, the adults exhibited high diffusion and were spread throughout the wooden container due to the high movement speed (Fig. 5). However, when the temperature was low (10 and 5°C), a high density of adults was found near the center location, close to their introduction area (Fig. 6). For instance, at 30°C more than 40% adults were recovered at the bottom layer (layer 7) (Fig. 5, Table 7) and the adults were distributed evenly across the different areas (Fig. 5, Table 7). Therefore, the adults had no preference for different areas. At 10°C, a higher density of adults was recovered in the center location in all layers (Fig. 6, Table 7). These results confirmed that the mobility of *C. ferrugineus* adults was significantly reduced by low temperatures.

**Table 7.** Recovered adult densities of *Cryptolestes ferrugineus* in different areas in the same layer at the same tested conditions

Layer	Time (h)^λ^	Center	Middle	Periphery	Tukey test values	
					*F*	*P*
30°C^µ^						
1^γ^	1	0.1 ± 0.1 ^a^	0 ^a^	0 ^a^	1.000	0.422
	2	0.1 ± 0.2 ^a^	0 ^a^	0 ^b^	1.000	0.422
	3	0.2 ± 0.2 ^a^	0.3 ± 0.0 ^a^	0.0 ± 0.1 ^a^	2.385	0.173
	24^β^	0 ^a^	0.1 ± 0.1 ^a^	0.1 ± 0.1 ^a^	1.245	0.353
2^γ^	1	0.4 ± 0.1 ^a^	0.1 ± 0.1 ^a^	0.0 ± 0.01 ^b^	21.800	0.002
	2	0.2 ± 0.3 ^a^	0.0 ± 0.1 ^a^	0 ^b^	0.833	0.480
	3	0.2 ± 0.3 ^a^	0 ^a^	0.0 ± 0.1 ^a^	0.840	0.477
	24^β^	0.1 ± 0.1 ^a^	0 ^a^	0.2 ± 0.2 ^a^	1.361	0.326
3^γ^	1	0.9 ± 0.5 ^a^	0.0 ± 0.1^b^	0 ^b^	10.290	0.012
	2	0.3 ± 0.4 ^a^	0.1 ± 0.1 ^a^	0.0 ± 0.1 ^a^	0.735	0.518
	3	0.3 ± 0.2 ^a^	0.0 ± 0.1 ^a^	0.0 ± 0.1 ^a^	3.036	0.123
	24^β^	0 ^a^	0.1 ± 0.1 ^a^	0.2 ± 0.1 ^a^	3.523	0.097
4^γ^	1	4.5 ± 2.1 ^a^	0.0 ± 0.1 ^b^	0.1 ± 0.1 ^b^	13.370	0.006
	2	4.4 ± 1.8 ^a^	0.1 ± 0.1 ^b^	0.1 ± 0.1 ^b^	16.890	0.003
	3	4.2 ± 1.5 ^a^	0.1 ± 0.1 ^b^	0.0 ± 0.1 ^b^	21.850	0.002
	24^β^	0.9 ± 0.7 ^a^	0.0 ± 0.1 ^a^	0.1 ± 0.1 ^a^	4.555	0.063
5^γ^	1	0.4 ± 0.5 ^a^	0 ^a^	0.1 ± 0.1^a^	1.880	0.232
	2	0.3 ± 0.3 ^a^	0.1 ± 0.1 ^a^	0.2 ± 0.2 ^a^	0.611	0.573
	3	0.3 ± 0.2 ^a^	0.2 ± 0.2 ^a^	0.1 ± 0.1 ^a^	1.754	0.251
	24^β^	0 ^a^	0 ^b^	0.2 ± 0.1 ^b^	7.618	0.023
6^γ^	1	0.4 ± 0.2 ^a^	0.1 ± 0.1 ^a^	0.1 ± 0.1 ^a^	4.092	0.076
	2	0.6 ± 0.5 ^a^	0.1 ± 0.1 ^a^	0.3 ± 0.3 ^a^	1.299	0.340
	3	0.6 ± 0.7 ^a^	0.3 ± 0.3 ^a^	0.2 ± 0.1 ^a^	0.530	0.614
	24^β^	0.4 ± 0.5 ^a^	0.2 ± 0.2 ^a^	0.5 ± 0.3 ^a^	0.467	0.648
7^γ^	1	3.1 ± 1.3 ^a^	0.6 ± 0.4^b^	0.7 ± 0.3 ^ab^	8.938	0.016
	2	2.9 ± 0.3 ^a^	0.5 ± 0.1 ^b^	0.6 ± 0.2 ^b^	88.610	<0.001
	3	3.0 ± 0.7 ^a^	0.5 ± 0.1 ^b^	0.7 ± 0.5 ^b^	27.490	0.001
	24^β^	2.0 ± 0.3 ^a^	0.7 ± 0.5 ^b^	1.8 ± 0.2 ^a^	9.853	0.013
20°C ^µ^						
1^γ^	1	0.1 ± 0.1 ^a^	0 ^a^	0 ^a^	1.000	0.422
2^γ^	1	0.1 ± 0.2^a^	0 ^a^	0 ^a^	1.000	0.422
3^γ^	1	0.1 ± 0.2 ^a^	0.0 ± 0.1 ^a^	0 ^a^	0.742	0.515
4^γ^	1	5.6 ± 2.3 ^a^	0 ^b^	0 ^b^	17.550	0.003
5^γ^	1	0.7 ± 0.4 ^a^	0.0 ± 0.1 ^b^	0 ^b^	7.764	0.022
6^γ^	1	0.8 ± 0.4 ^a^	0.0 ± 0.01^b^	0.0 ± 0.1^b^	9.314	0.015
7^γ^	1	2.8 ± 1.0 ^a^	1.0 ± 0.6 ^b^	0.5 ± 0.3 ^b^	9.470	0.014

^β^Data used from Bharathi et al. (2022a).

^µ^Grain temperature.

^λ^Movement period.

^γ^The alphabets a and b in the same row indicate significantly different mean values in a layer under the same tested condition.

Tukey’s test at a 0.05 confidence level was used to conduct these comparisons with degrees of freedom (df) = 2 for each row comparison.

## Discussions

### Effect of Temperature, Time, and Grain Bulk on Movement and Distribution

The 3-D movement and distribution of *T. castaneum* and *C. ferrugineus* adults were influenced by both temperature and movement period. Even though both adults moved in both horizontal and vertical directions, the movement speeds of both species were significantly influenced by temperature (Table 1). Their vertical and horizontal movements decreased noticeably as the temperature reduced from 30 to 5°C (Figs. 3–6). They reached the boundary of the wooden container due to the high movement speed at higher temperatures within 1 h.

In 1-D study by Jian et al. (2004a), which investigated the movement of *C. ferrugineus* within stored wheat using a 10 × 10 × 100 cm grain column. Both vertical and horizontal directions were analyzed under linear and dynamic temperature gradients ranging from 25 to 35°C. The study involved four insect densities and movement periods of 12, 24, 72, and 144 h. The results indicated that adults moved at speeds exceeding 10.8 m/d in the vertical direction and up to 6 m/d in the horizontal direction within the grain column. Our 3-D study showed that the adults exhibit movement speeds of more than 7.2 m/d in both vertical and horizontal directions at higher temperatures of 30 and 20°C. While the vertical movement speed in our study is similar to the 1-D study, the higher horizontal speed observed in our results could be due to the size of the grain container. In the 1-D study, the adults had limited space, whereas inside the 3-D setup they had more space to move in any direction. Therefore, insect movement speed in the vertical direction was not influenced by the size and dimension of the container, while the horizontal movement was limited by the size and dimension of the container. This observation is important for grain storage management, as the measured insect movement under laboratory conditions can be applied to grain storage bins of any shape and size.

The recovery rate of *C. ferrugineus* adults from the introduced cube at 20 and 30°C in 1 h ranged from 35 to 42%. The aggregation behavior observed in the *C. ferrugineus* can be attributed to pheromone production during the acclimation period. This is supported by Bharathi et al. (2022a), who reported recovery rates in the introduced cube of their 24 h 3-D studies in wheat with 12.5% moisture content at 20 and 30°C for *C. ferrugineus* were between 12% and 13%. The difference in recovery rates from our results could be due to the higher moisture content and shorter movement periods because insects prefer 14.5% moisture more than that of 12.5% (Anukiruthika et al. 2023). Similarly, in a study by Halliday and Blouin-Demers (2016) investigating the Ideal Free Distribution (IFD) of *T. castaneum* adults, it was found that mixed-sex groups of adults aggregated more than female-only groups due to the secretion of aggregation pheromones. This aggregation behavior can cause a deviation from their IFD. These findings have practical implications for pest control strategies, as they explain that insect movement and distribution in grain bulk are influenced by many factors (such as temperature and movement period studied in this article) and the interaction among insects (Jian et al. 2009), which results in different distribution patterns in grain bins.

### Distribution Pattern and Movement Behavior

In our 3-D study, we observed that at 30°C over a 24-h period, *C. ferrugineus* adults exhibited a 20% higher downward movement than *T. castaneum*. Jian et al. (2005b) explained the more downward movement of *C. ferrugineus* compared to *T. castaneum* was due to the drift effect caused by their smaller body size relative to *T. castaneum*. Most researchers categorized this downward movement as a geotaxis behavior (Jian et al. 2002, 2005a, 2006, Bharathi et al. 2022b). This suggests that body size and geotactic responses play significant roles in the vertical movement patterns of these pests under different environmental conditions. In a laboratory study using a modified dispersal apparatus consisting of interconnected containers and tubes to track insect movement, it was found that *T. castaneum* showed a greater tendency to climb up in high-density conditions. It was suggested that this dispersal behavior could be influenced by interactions such as repulsion from conspecifics of the same sex (Arnold et al. 2017). In our study, *T. castaneum* adults showed more upward movement than *C. ferrugineus* adults. Specifically, in 2 h at 30°C, 17% more *T. castaneum* adults were recovered in the top layers than that of *C. ferrugineus*. This difference becomes even more prominent at the temperature of 20°C in 1 h, where the upward movement of *T. castaneum* surpasses the rusty grain beetle movement by ~23%. In our current study, since we did not differentiate adults by sex, it is likely that high densities of individuals of the same sex could exist, potentially leading to increased repulsion and thereby promoting dispersal among the adults. Similar findings have been reported in the literature (Surtees 1963, Jian et al. 2005b, 2012, Bharathi et al. 2023b).

The random horizontal movement behavior observed at high temperatures, specifically at 20°C for 1 h and at 30°C for durations of 1, 2, 3, and 24 h in both species contributes to the diffusion pattern within the 3-D setup. These findings are consistent with previous 1-D and 2-D studies, which demonstrated that the randomness in their movement is a key factor in creating diffusion patterns (Jian et al. 2004b, 2007). This random movement results in a spread of insects throughout the grain mass, preventing the formation of clusters and reducing the competition for resources. Although insects possess strong olfactory senses, the physical structure of bulk grain poses significant challenges to their communication. Within the dense and compact grain mass, the diffusion of pheromones is restricted, and the concentration of these chemical signals is reduced, making it difficult for insects to detect and respond to them effectively (Obeng-Ofori and Coaker 1990). This is proven in a laboratory study by Currie et al. (2020) who examined the attractiveness of pheromones in *C. ferrugineus* adults with and without the presence of grain inside an apparatus monitored by an infrared camera. It was found that within the grain, there was no attraction between conspecifics at low insect densities. The researchers attributed this lack of attraction to the absorption of pheromones by the grain and the absence of air currents to disperse the pheromones effectively. Similarly, insects that might use acoustic signals for communication are also compromised in bulk grain environments. As these signals travel through the grain, they tend to weaken, and the physical structure of the grain acts as a barrier and reduces the range and clarity (Mankin et al. 2011). Grain kernels also obstruct their line of sight, resulting in a lack of visual cues. So, consequently, the beetles move in a random pattern as they navigate through the obstructed environment (Jian et al. 2009). This consistency in behavior across different studies (Jian et al. 2002, 2007, Bharathi et al. 2022a) highlights the insect’s tendency for random dispersal in a stable and uniform environment.

### Different Movement Behaviors Between Different Species

The results of this 3-D study indicated distinct movement behaviors for *T. castaneum* and *C. ferrugineus* adults at 30°C. The *T. castaneum* adults initially move upwards, then downwards, and finally move horizontally. In contrast, *C. ferrugineus* adults start by moving downwards, then upwards, and followed by horizontal movement. This suggests that each species has unique behavioral responses to temperature, influenced by their physiological and ecological adaptations (Anukiruthika et al. 2021). In a study by Jian et al. (2005b), the movement of *T. castaneum* was analyzed in both horizontal and vertical wheat and corn columns with and without temperature gradients. They discovered that over 90% of *T. castaneum* remained in their introduced area for 6 d within the wheat grain column when temperatures were uniform. This high aggregation rate was attributed to the smaller granular size of the wheat bulk, which made it difficult for the beetles to move through the grain. Their large body size relative to the wheat pores created a physical barrier that hindered their ability to disperse into the bulk, resulting in a higher concentration of beetles in the insect introduction area. A recent study (Pointer et al. 2024) on the dispersal behavior of *T. castaneum* revealed that individuals with high dispersal tendencies exhibited several distinct traits. It was found that the high-dispersal beetles were significantly more active, traveled in straighter paths, and were more frequently found exposed on the surface of their food. This increased surface activity was attributed to boldness. In addition, these beetles tended to be larger in size compared to their less-dispersive counterparts. Pointer et al. (2024) suggested that the dispersal behavior in *T. castaneum* was not an isolated trait but part of a broader characteristics, referred to as the dispersal syndrome. This concept highlights that dispersal tendencies are closely intertwined with behavioral, physiological, and morphological traits. These findings partially explain the difference between species, and their biological behavior such as food preference, predator–prey relationships, and mating behavior also influence their movement. These physical and biological factors together shaped the movement and distribution patterns among species.

A study on the movement and distribution of *C. ferrugineus* and *T. castaneum* adults was conducted in 300 t of wheat in a 10-m diameter bin from September 2019 to October 2021 in Winnipeg, Canada (Bharathi et al. 2023b). The results showed that *C. ferrugineus* adults, released at the top layer at the start of the experiment, initially moved downwards and then spread horizontally. In contrast, most of the *T. castaneum* adults remained near their introduction point but some were also found throughout the bin. Both adult species were found near the warmer regions of the grain bin. However, in our study, both species exhibited higher densities at the periphery of the bottom layer (layer 7). This might be caused by the physical boundary limitation of the wooden container in our study. In a study conducted by Collins and Conyers (2009) on the movement of *Oryzaephilus surinamensis* (Linnaeus) and *Sitophilus granarius* (L.) adults inside a bin with a surface area of 9 m² and a depth of 4 m filled with 30 t of wheat, it was reported that *O. surinamensis* adults exhibited an upward movement pattern without horizontal dispersion, while *S. granarius* adults showed less upward movement. Another study on the movement and distribution of *Sitophilus zeamais* by Zhang et al. (2020) in a bin with a diameter of 1.2 m and a depth of 1.2 m filled with 1 t of wheat reported that the adults demonstrated less upward and more downward movement in the vertical direction. The observed differences in movement behavior among various insect species can be attributed to factors such as their point of introduction into the grain bulk, the presence of temperature and moisture gradients within the storage environment, and the factors mentioned in the previous paragraph. These findings highlight the species-specific movement and distribution patterns in stored grain environments.
